# Telomere length and mitochondrial DNA copy number in bipolar disorder: identification of a subgroup of young individuals with accelerated cellular aging

**DOI:** 10.1038/s41398-022-01891-4

**Published:** 2022-04-01

**Authors:** L. Spano, B. Etain, M. Meyrel, V. Hennion, G. Gross, J-L. Laplanche, F. Bellivier, C. Marie-Claire

**Affiliations:** 1 Université de Paris, INSERM UMR-S 1144, Optimisation Thérapeutique en Neuropsychopharmacologie, OTeN, F-75006 Paris, France; 2 Département de Psychiatrie et de Médecine Addictologique, Hôpitaux Lariboisière-Fernand Widal, GHU APHP.Nord – Université de Paris, Paris, F-75010 France; 3 Pôle Hospitalo-Universitaire de Psychiatrie d’Adultes et d’Addictologie du Grand Nancy, Centre Psychothérapique de Nancy, Laxou, France; 4 Université de Lorraine, Vandoeuvre-lès-Nancy, France; 5 Département de Biochimie et Biologie Moléculaire, DMU BioGeM, Hôpitaux Lariboisière-Fernand Widal, GHU APHP.Nord – Université de Paris, Paris, F-75010 France

**Keywords:** Bipolar disorder, Biomarkers

## Abstract

The 10–15-years decrease in life expectancy observed in individuals with bipolar disorder (BD) has been linked to the concept of accelerated cellular aging. Telomere length (TL) and mitochondrial DNA copy number (mtDNAcn) have been proposed as markers of cellular aging and comparisons between individuals with BD and healthy controls (HC) sometimes led to conflicting results. Previous studies had moderate sample sizes and studies combining these two markers into a single analysis are scarce. Using quantitative polymerase chain reaction, we measured both TL and mtDNAcn in DNA (peripheral blood) in a sample of 130 individuals with BD and 78 HC. Regression analyses, receiver operating characteristic (ROC), and clustering analyses were performed. We observed significantly lower TL and mtDNAcn in individuals with BD as compared to HC (respective decrease of 26.5 and 35.8%). ROC analyses showed that TL and mtDNAcn highly discriminated groups (AUC = 0.904 for TL and AUC = 0.931 for mtDNAcn). In the whole population, clustering analyses identified a group of young individuals (age around 36 years), with accelerated cellular aging (both shorter TL and lower mtDNAcn), which consisted mostly of individuals with BD (85.5%). The subgroup of patients with young age but accelerated aging was not characterized by specific clinical variables related to the course of BD or childhood maltreatment. However, patients in this subgroup were more frequently treated with anticonvulsants. Further characterization of this subgroup is required to better understand the molecular mechanisms and the risk factors of accelerated cellular aging in BD.

## Introduction

Bipolar disorder (BD) is a chronic psychiatric illness that affects at least 1% of the general population and is a leading cause of burden and societal costs [1]. A decrease of about 10–15 years in life expectancy has been observed in individuals with BD [2] as compared to the general population, this being not only explained by an increased prevalence of suicide, but also by comorbid somatic diseases [3]. Among all natural causes of premature death, age-related diseases such as cardiovascular and metabolic disorders, are the most common explanations for the reduction of life expectancy, and this further suggests that BD may be characterized by accelerated aging [3, 4]. Over the last decade, several markers of cellular aging, such as telomere length (TL) and the copy number of mitochondrial DNA (mtDNAcn), have been studied in individuals with BD as compared to healthy controls (HC) [5].

Telomeres are DNA repeat sequences found at the end of chromosomes and protect chromosomes against degradation and fusion [6]. Due to the incomplete DNA replication at each cell division, the length of telomeres decreases with age, making it a marker of aging [6, 7]. TL has been the most widely studied marker of cellular aging in BD and in most available studies, a significant reduction in TL was observed in individuals with BD as compared to controls (even after adjustment for any difference in chronological age) [8–10]. These results were further confirmed by a recent meta-analysis of ten studies including 579 individuals with BD and 551 controls (effect size g = −0.54, 95% CI = −0.84 to −0.28, *p* < 0.001) [11]. Of note, a previous meta-analysis including seven studies with 633 BD and 482 controls [12] did not find any difference for TL (Effect size g = −0.012, 95% CI = −0.418 to 0.393, *p* = 0.95). Therefore, as recently discussed in a comprehensive review [5], the results obtained with TL may still be considered as controversial.

The number of copies of mitochondrial DNA is another well-described marker of cellular aging, however much less studied in BD [13]. With age, a dysfunction of the respiratory chain in mitochondria with an accumulation of damage and mutations in mtDNA is observed. This leads to a decrease of mtDNAcn that therefore negatively correlates with age [14]. The number of studies of mtDNAcn in BD is still limited and the results remain inconsistent [5]. Indeed, three studies found a reduction in mtDNAcn in individuals with BD as compared to controls [13, 15, 16], while two studies found no difference [17, 18] and one study found an increase of mtDNAcn in individuals with BD [19].

The inconsistent results in the literature may result from the small sample sizes being used so far since most studies were performed in less than 100 participants, but also the heterogeneity of participants’ mood states at inclusion (euthymic, depressed, and manic patients). Moreover, only one previous study has investigated both markers of aging at the same time. The authors found no difference in TL between BD individuals and controls and higher levels of mtDNAcn in BD, however did not combine the two markers [19]. Finally, the associations between markers and potential determinants or risk factors remain unclear. Several risk factors such as obesity, smoking, and childhood maltreatment have been suggested to be associated with premature aging in BD [20–24], while exposure to lithium has been suggested to be “protective” against accelerated aging. These results are not consistently replicated from one study to another [5].

Given the need of studies investigating both TL and mtDNAcn, we investigated these markers in a cohort of individuals with BD as compared to healthy controls (HC). We also studied the associations between these markers and the characteristics of BD in order to better identify those individuals with BD who are more at risk of accelerated cellular aging.

## Materials and methods

### Participants

The sample consisted of 130 individuals with BD type I and II according to DSM-IV criteria (Diagnostic and Statistical Manual of Mental Disorders—fourth edition) [25]. Participants were recruited in one university-affiliated psychiatric department in Paris (France). Inclusion criteria for this study were as follow: Caucasian origin; age over 18 years; euthymic for at least 3 months (as defined by the absence of any major mood episode and no changes in current psychotropic medication in the last 3 months before inclusion) and scores below 8 at the Montgomery–Asberg depression rating scale [26] and the Young Mania Rating Scale [27] at inclusion. The SCID (Structured Clinical Interview for DSM Disorders) was used to confirm the diagnosis of BD and to collect lifetime information related to the course of BD. Childhood traumatic events were assessed with the French validated version [28] of the Childhood Trauma Questionnaire [29]. Healthy controls (*n* = 78), with no personal or familial history of mood disorders nor suicidal behavior, were also recruited in this study. The clinical assessment of controls was performed with the SCID.

For all participants, we collected information about age, sex, Body Mass Index (BMI), smoking status, MADRS, and YMRS scores at inclusion. Current alcohol misuse and past or current substance misuse were exclusion criteria. However, past alcohol misuse was not an exclusion criterion and was considered in the analyses. For Individuals with BD, we collected information about the age of onset of BD (age at the first depressive, (hypo)manic or mixed episode according to DSM-IV criteria), duration of the illness, a number of major mood episodes, family history of BD, current prescribed mood stabilizers (lithium, anticonvulsants, atypical antipsychotics), and antidepressants at inclusion.

Written informed consent was obtained from all participants. This research protocol (Clinical Trials Number NCT02627404) was approved by the French medical ethics committee (Comité de Protection des Personnes (CPP)—IDRCB2008_AO1465_50 VI – Pitié-Salpêtrière 118–08) and carried out according to the approved guidelines.

### TL and mtDNAcn measurements

A blood sample was collected at inclusion. Genomic DNA was extracted from peripheral blood samples using the automated Maxwell 16 DNA Purification Instrument and the dedicated Maxwell^®^ RSC Blood DNA Kit (Promega). Real-time quantitative polymerase chain reaction (PCR) was used to measure telomere length and mitochondrial DNA copy number. It was carried out on a CFX384 Touch Real-Time PCR Detection System (Biorad). Beta-hemoglobin gene was used as a single-copy standard to obtain a relative quantification of TL and mtDNAcn. All PCRs were performed in a reaction volume of 10 μL containing 5 μL of 2x SsoAdvanced Universal SYBR Green Supermix (Biorad), 25 ng of DNA from whole blood and 300 nmol/L of each primer. The following sequences were used for TL 5′-CGGTTTGTTTGGGTTTGGGTTTGGGTTTGGGTTTGGGTT-3′ (TL-F) and 5′-GGCTTGCCTTACCCTTACCCTTACCCTTACCCTTACCCT-3′ (TL-R); mtDNAcn 5′-CATCTGGTTCCTACTTCAGGG-3′ (mtDNAcn-F) and 5′-TGAGTGGTTAATAGGGTGATAGA-3′ (mtDNAcn-R) and beta-hemoglobin measurements 5′-GCTTCTGACACAACTGTGTTCACTAGC-3′ (beta_hemoglobin-F) and 5′-CACCAACTTCATCCACGT-3′ (beta_hemoglobin-R) as previously described [30]. A standard curve with serial dilutions of reference DNA was used to analyze the efficiency of primers. PCR parameters were as follows: an initial heating step of 98 °C for 3 min followed by 40 cycles of 98 °C for 15 s and 60 °C for 30 s. At the end of amplification, a melting curve was used to verify the specificity of primers. Delta delta Cq calculation method was used to obtain relative TL and mtDNAcn [31]. All samples were assessed in triplicate.

### Statistical analysis

JAMOVI [32] and JASP [33] softwares were used to perform statistical analysis. All results were considered as significant when *p* value <0.05 and no correction for multiple testing was applied in this exploratory study. Mann–Whitney (for continuous variables) and Chi2 tests (for categorical variables) were used to compare individuals with BD and controls for socio-demographic variables. Spearman correlation tests were used to investigate the associations between age, TL, and mtDNAcn.

To study the differences for TL and mtDNAcn between BD and HC groups, two separate linear regressions were performed with an adjustment for age, sex, BMI, current tobacco use, MARDS, and YMRS scores. Second, two logistic regressions were performed with clinical status as the dependent variable (BD versus HC), and either TL or mtDNAcn as independent variables, with an adjustment for age, sex, BMI, current tobacco use, MADRS, and YMRS scores. The sensitivity and specificity of these models were estimated using the receiver operating curve (ROC) and a calculation of the area under the curve (AUC).

Finally, we performed a K-means clustering analysis using Lloyd–Forgy algorithm with age, TL, and mtDNAcn as classification variables in the whole sample (BD and HC). Analysis of the association between clinical variables and markers of aging in each cluster was performed using Kruskal–Wallis test (for continuous variables) or Chi^2^ test (for categorical variables), with post hoc comparisons between groups.

## Results

### Sample characteristics

Characteristics of individuals with BD (*n* = 130) and HC group (*n* = 78) are shown in Table 1. The two groups did not differ for sex-ratio (*p* = 0.799). Individuals with BD were older (*p* = 0.018), had a higher BMI (*p* = 0.005) and were more frequently current smokers (*p* = 0.017) than HC. As expected, individuals with BD also had higher MADRS (*p* < 0.001) and YMRS (*p* < 0.001) scores than HC. In the BD group, a majority of individuals had been diagnosed with BD type 1 (75.4%).

**Table 1:** Characteristics of individuals with bipolar disorders and healthy controls group.

Variables	BD (*n* = 130)	HC (*n* = 78)	*p* value
Age (years), mean ± SD	44.3 ± 12.9	40.8 ± 15.1	0.018^a^
Sex (female), *n* (%)	76 (58.5%)	47 (60.3%)	0.799^b^
BMI (kg/m^2^), mean ± SD	25.7 ± 4.58	23.9 ± 3.76	0.005^a^
MADRS, mean ± SD^c^	2.46 ± 2.79	0.22 ± 0.89	<0.001^a^
YMRS, mean ± SD^c^	0.78 ± 1.59	0.04 ± 0.25	<0.001^a^
Tobacco (yes), *n* (%)^d^	58 (45.4%)	18 (28.6%)	0.017^b^

*BD* bipolar disorder, *HC* healthy controls, *BMI* body mass index, *MADRS* Montgomery–Asberg depression

rating scale, *YMRS* Young mania rating scale.

^a^Mann–Whitney test.

^b^chi^2^ test.

^c^1 missing value in BD.

^d^1 missing value in HC.

### Comparisons of BD and HC groups for TL and mtDNAcn

TL and mtDNAcn were positively correlated in individuals with BD (rho = 0.221; *p* = 0.011) but not in HC (rho = 0.044; *p* = 0.701). TL was negatively correlated with age in both BD and HC groups (respectively rho = −0.362 and rho = −0.392; *p* < 0.001), but mtDNAcn was not correlated with age (rho = −0.160, *p* = 0.069 for BD and rho = 0.101, *p* = 0.377 for HC) (Fig. S1).

**Fig. 1:**
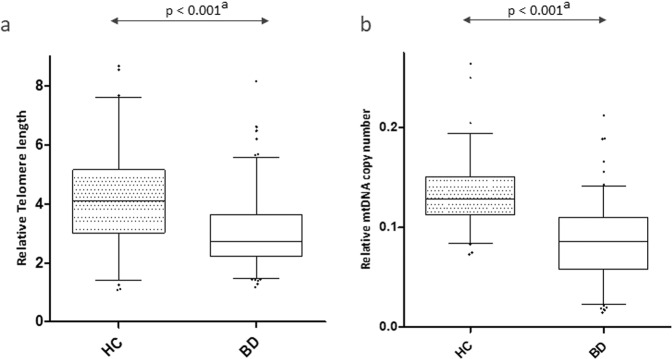
Telomere length and mtDNA copy number differences between individuals with BD and healthy controls. Box plots representing relative Telomere length (**a**) and relative mtDNA copy number (**b**) in 130 BD and 78 HC. BD bipolar disorder, HC healthy controls. ^a^*p* values indicate differences between BD and HC after adjustment for age, sex, BMI, tobacco, MADRS, and YMRS (linear regressions).

Univariate analyses (Mann–Withney tests) showed that TL and mtDNAcn were both lower in BD as compared to HC (reduction of 26.5% for TL *p* < 0.001 and reduction of 35.8% for mitDNAcn *p* < 0.001). These differences between groups remained significant (both *p* values <0.001) after adjustment for age, sex, BMI, MADRS scores, YMRS scores, and smoking status in linear regressions (Fig. 1 and Table S1).

**Fig. 2:**
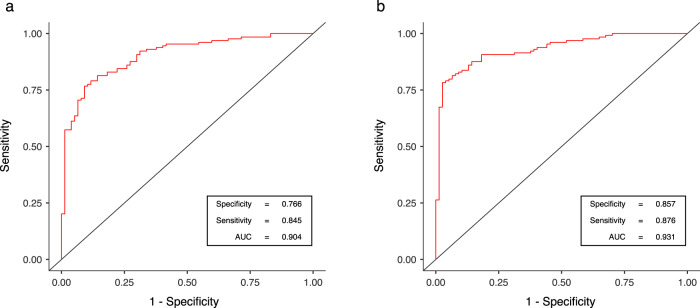
ROC curves from logistic regression of TL and mtDNAcn between BD and HC. Logistic regressions (BD versus HC) were performed with either TL (**a**) or mtDNAcn (**b**), with an adjustment for covariates (age, sex, BMI, current tobacco use, MADRS, and YMRS).

Logistic regressions (BD versus HC) adjusted for potential covariates (Table S2) and ROC analyses (Fig. 2) indicated that each marker significantly discriminated BD from HC (for TL: specificity = 0.766; sensitivity = 0.845; AUC = 0.904 and for mtDNAcn: specificity = 0.857; sensitivity = 0.876; AUC = 0.931).

### Cluster analysis based on age and markers of aging

**Fig. 3:**
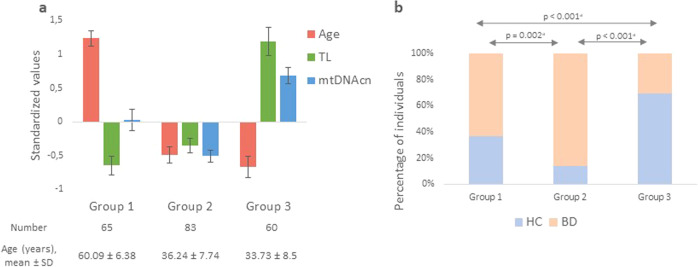
Clustering of pooled BD and HC groups with age, TL, and mtDNAcn as clustering variables. Standardized values of age, TL, and mtDNAcn (**a**) are shown for each group created by the clustering analysis. Histograms (**b**) represent the percentage of HC and BD in each cluster. ^a^Chi^2^ tests.

In order to identify potential subgroups of individuals with accelerated aging, a k-means clustering analysis was performed in the pooled sample of BD and HC groups using TL, mtDNAcn, and age as classification variables. As shown in Fig. 3, we identified three subgroups of individuals. Group 1 (*N* = 65) consisted of individuals with a mean age around 60 years, short TL, and low mtDNAcn (Fig. 3a). Although of similar mean ages, Group 2 (*N* = 83, mean age around 36 years) and Group 3 (*N* = 60, mean age around 33 years) displayed opposite TL and mtDNAcn patterns. TL and mtDNAcn were decreased in Group 2 while preserved in Group 3 (Fig. 3a). Therefore, Group 2 corresponded to a subgroup of individuals who were young in age but with already altered biomarkers of cellular aging.

The distribution of individuals with BD and HC significantly differed in groups 2 and 3 (*p* < 0.001, Fig. 3b). Indeed, individuals with BD represented 85.5% of individuals in Group 2 (young age/altered biomarkers of cellular aging), but only 30% in Group 3 (young age/preserved biomarkers of cellular aging) (Fig. 3b).

We finally compared the three groups of individuals with BD for variables related to the course of BD and current medications (Table 2). As expected, the three groups significantly differed for age, TL, and mtDNAcn which were the variables used for the clustering analysis. Group 1 was significantly older (*p* < 0.001) than the other two groups. Group 2 (young age/accelerated aging) had intermediate values for TL (*p* < 0.001), but already mtDNacn at the level of the oldest group (*p* < 0.001). Group 1 (oldest group) was further characterized by a later age at onset and a longer duration of the illness as compared to the other groups (both comparison, *p* < 0.001), which was expected given their older age. Group 2 (young age/accelerated aging) had the highest densities of depressive and manic episodes. However, these densities were different only when compared to Group 1 (old age/accelerated aging), but not to Group 3 (young age/no accelerated aging). Even if individuals in Group 3 tend to have more frequent use of lithium than Group 2 (72.2 vs 65.2%), the only characteristic that differed significantly between Group 2 (young age/accelerated aging) and Group 3 (young age/no accelerated aging) was a more frequent current use of anticonvulsants in Group 2 (respectively 56.5 versus 22.2%; *p* = 0.010) (Table 2).

**Table 2:** Comparisons between the three groups resulting from the clustering analyses (individuals with BD only).

Variables	Cluster 1 (*n* = 41)	Cluster 2 (*n* = 71)	Cluster 3 (*n* = 18)	*p* value global	Group comparisons
Age (years), mean ± SD	59.8 ± 6.31	37.1 ± 7.76	37.0 ± 8.74	<0.001^a^	1 > 2 = 3 (*p* < 0.001)
TL, mean ± SD	2.37 ± 0.73	2.90 ± 0.76	5.33 ± 1.07	<0.001^a^	1 < 2 < 3 (*p* < 0.001)
mtDNAcn, mean ± SD	0.08 ± 0.04	0.08 ± 0.03	0.12 ± 0.04	<0.001^a^	3 > 1 = 2 (*p* < 0.001)
Sex (female), *n* (%)	22 (53.7%)	41 (57.7%)	13 (72.2%)	0.405^b^	
BMI (kg/m^2^), mean ± SD	26.7 ± 4.37	25.0 ± 4.82	25.4 ± 3.24	0.062^a^	
Tobacco (yes), *n* (%)	14 (34.1%)	34 (49.3%)	10 (55.6%)	0.194^b^	
Past alcohol misuse (yes), n (%)	10 (24.4%)	22 (30.9%)	5 (27.7%)	0.711^b^	
MADRS, mean ± SD	2.32 ± 2.51	2.52 ± 3.11	2.41 ± 2.21	0.935^a^	
YMRS, mean ± SD	0.87 ± 1.85	0.72 ± 1.40	0.47 ± 0.80	0.988^a^	
Lifetime suicide attempts (yes), *n* (%)	16 (39.0%)	24 (33.8%)	6 (33.3%)	0.840^b^	
BD type 1, *n* (%)	15 (36.6%)	14 (19.7%)	3 (16.7%)	0.096^b^	
Age of onset (years), mean ± SD	33.0 ± 11.8	23.6 ± 6.38	23.0 ± 9.65	<0.001^a^	1 > 2 (*p* < 0.001); 1 > 3 (*p* = 0.004); 2 = 3
Duration of illness (years), mean ± SD	26.3 ± 11.8	13.8 ± 7.49	14.0 ± 7.77	<0.001^a^	1 > 2 = 3 (*p* < 0.001)
Density of number of depression, mean ± SD	0.30 ± 0.35	0.51 ± 0.66	0.38 ± 0.23	0.034^a^	1 < 2 (p = 0.016); 1 < 3 (*p* = 0.045); 2 = 3
Density of number of mania, mean ± SD	0.07 ± 0.14	0.18 ± 0.21	0.16 ± 0.15	<0.001^a^	1 < 2 = 3 (*p* < 0.001)
CTQ total score, mean ± SD	41.5 ± 12.8	39.5 ± 13.1	40.8 ± 13.5	0.571^a^	
Family history of BD (yes), *n*(%)	15 (36.6%)	23 (32.4%)	7 (38.9%)	0.831^b^	
Li (yes), *n* (%)	28 (70.0%)	45 (65.2%)	13 (72.2%)	0.795^b^	
AC (yes), *n* (%)	19 (47.5%)	39 (56.5%)	4 (22.2%)	0.034^b^	2 > 3 (*p* = 0.010)
APA (yes), *n* (%)	10 (25.0%)	23 (33.8%)	8 (44.4%)	0.325^b^	
AD (yes), *n* (%)	17 (42.5%)	19 (27.5%)	4 (22.2%)	0.177^b^	

*TL* telomere length, *mtDNAcn* mitochondrial DNA copy number, *BMI* body mass index, *MADRS* Montgomery-Asberg depression rating scale, *YMRS* Young mania rating scale, *CTQ* childhood trauma questionnaire, *Li* lithium, *AC* anticonvulsant, *APA* antipsychotic, *AD* antidepressant.

^a^Kruskal–Wallis test.

^b^chi^2^ test.

## Discussion

Accelerated cellular aging has been proposed as one of the mechanisms being involved in the reduction of life expectancy observed in BD. Our study confirmed, in a well-characterized sample of euthymic individuals with BD, a reduction of TL as compared to healthy controls. We also demonstrated a significant decrease in mtDNAcn in individuals with BD as compared to HC. This study adds to the existing literature since a clustering analysis identified for the first time a subgroup of young individuals, that consisted mainly of individuals with BD, with already altered TL and mtDNAcn. Comparisons between subgroups of patients of similar young age but with dissimilar markers of cellular aging showed no major differences for clinical variables related to BD presentation, except for a difference in terms of current use of anticonvulsants.

Although some inconsistent results may exist in the literature for TL, the most recent meta-analysis concluded that individuals with BD present with shorter telomere as compared to HC [11]. Our results are therefore consistent with a premature telomere shortening in BD individuals. In addition, we found a reduction in mtDNAcn in the BD group. Interestingly though, this finding for mtDNAcn is consistent with those obtained in studies performed in samples of more than 100 individuals [13, 15, 16]. Other studies performed on smaller samples (*N* < 50) reported no differences or even an increase in mtDNAcn in individuals with BD [17–19]. Therefore, some of the discrepancies observed across studies about mtDNAcn might be due to a lack of statistical power.

We found a significant but moderate correlation between mtDNAcn and TL in the BD group suggesting that both markers are involved in accelerated cellular aging in BD and may decrease together. This result is consistent with those obtained in peripheral blood of 392 healthy adults study [34] or using leukocytes DNA from 129 healthy elderly women [35] but not with those from the only study exploring TL and mtDNAcn at the same time in BD [19]. This latter study found no correlation between these two markers in peripheral blood, however in a smaller sample (22 individuals with BD1, 16 of their siblings, and 20 controls) [19]. Therefore, further studies are still required to describe how these two markers may covary in individuals with BD (synchronous or asynchronous decrease).

Our study is the first to highlight the existence of a subgroup of young individuals with pronounced alterations of both markers of cellular aging that mainly consisted in individuals with BD. The identification of such a subgroup suggests that the mechanisms that lead to accelerated aging during the lifespan might intervene earlier in life in a subgroup of individuals with BD (as compared to controls). A few individuals with BD might have a “synchronous” cellular aging (a chronological age that “corresponds” to cellular age) and would be similar to HC, while most individuals with BD might present accelerated cellular aging starting at a relatively young age (and possibly even before the onset of BD). This result is consistent with most of the previous studies, except with two that found an accelerated aging phenotype only in older patients, therefore leading to the hypothesis of a cumulative effect of life stressors on this accelerating aging processes [19, 36]. These discrepant results may be explained for instance by disparities in the biomarkers of cellular aging studied (TL and mtDNAcn versus epigenetic age) [19, 37].

The imbalance between proportions of BD and HC between cluster 2 and cluster 3 deserves some comments. One obvious interpretation is that individuals with BD have been much more exposed to environmental risk factors of accelerated aging that intervene early in life or even during pregnancy. This sample was only characterized for childhood maltreatment which levels did not differ between clusters. We did not have any information regarding other early environmental risk factors in this sample. These individuals may also have been less exposed to some protective factors against cellular aging. This imbalance might also be interpreted as a genetic predisposition, although this remains speculative. Nevertheless, cluster 2 does not include only individuals with BD, but also few controls. Hence, the existence of accelerated aging in a young population is not specific to BD, although much more frequent in this population. First, the existence of this subgroup requires replication before any conclusions regarding the mechanisms at stake. Second, since we could not identify major differences between clusters, it is not possible to formally conclude about the mechanism(s) involved (i.e., staging hypothesis [11], early life stress hypothesis [38], and the genetic hypothesis [39]), and in which specific subgroup these mechanisms might be at stake.

As a whole, the two groups of patients of young age but different for cellular aging markers appear very similar for their clinical presentation, with the exception of the current use of anticonvulsants. This could result from a lack of power due to the small size of Group 3. However, several trends may be noted: (i) although they had the same age, Group 2 seemed to have the highest densities of mood episodes (whatever the polarity being considered) than Group 3; (ii) individuals in Group 3 also tend to be more on lithium than Group 2 who seemed to be more on anticonvulsants which is consistent with the literature where individuals with BD taking lithium appear to have a longer telomere length than others. Some other variables that may be associated with cellular aging, such as suicide attempts [40] and substance use disorders [41] (current tobacco or past alcohol misuse for example) have been analysed in this study, but no significant differences were observed between subgroups. If replicated in larger and independent samples, the existence of such a subgroup would deserve further characterization to better identify which young individuals with BD might be more at risk for accelerated cellular aging.

Several limitations of this study deserve comments. First, all data were collected retrospectively, that may have biased some of our results. This may be the case for age at onset, number of episodes, and childhood maltreatment. Second, some variables were not collected such as cumulative duration of mood episodes or cumulative exposure to each type of mood stabilizer. Data related to somatic comorbidities, other psychiatric comorbidities (i.e., anxiety disorders), or blood cell type composition were also not available in this sample and therefore not taken into account in the analyses. This is a potential limitation since, for instance, several somatic comorbidities such as diabetes, cardiovascular disease, stroke, or cancer have been linked to accelerated aging [42, 43] and differences in telomere length have been observed between T-cells, B-cells, and monocytes [44, 45]. Third, despite our relatively large sample size compared to previously published studies in the field, we cannot definitively exclude some false-negative results. This may be the case when comparing the two youngest clusters (with dissimilar TL and mtDNAcn) with one group being composed of only 18 individuals. Finally, it would have been interesting to extend the analyses to other proposed markers of cellular aging such as epigenetic age, levels of inflammatory biomarkers, metabolic imbalance, or oxidative stress since they have been proposed as playing a role in the physiopathology of BD [46, 47] but also as associated with aging in other mental illnesses such as major depressive disorder [48] or schizophrenia [49].

To conclude, our study confirmed accelerated cellular aging in individuals with BD as compared to controls with a decrease in both TL and mtDNAcn in peripheral blood. We also evidenced the existence of a subgroup of young individuals with BD who were characterized by a pronounced discordance between their chronological age and their biological age as reflected by significant decreases in both TL and mtDNAcn. Further studies, in larger samples with additional clinical description, are required to replicate this clustering of individuals with BD based on their cellular aging markers, to better characterize this subgroup and thereby provide a better understanding of risk and protective factors of cellular aging in bipolar disorder.

## Supplementary information

Figure S1

Table S1

Table S2
