# Supplementary figures and images for: Telomere length and mitochondrial DNA copy number in bipolar disorder: identification of a subgroup of young individuals with accelerated cellular aging
Source: Transl Psychiatry. 2022 Apr 1;12:135. doi: 10.1038/s41398-022-01891-4 (PMC8975957; doi:10.1038/s41398-022-01891-4)

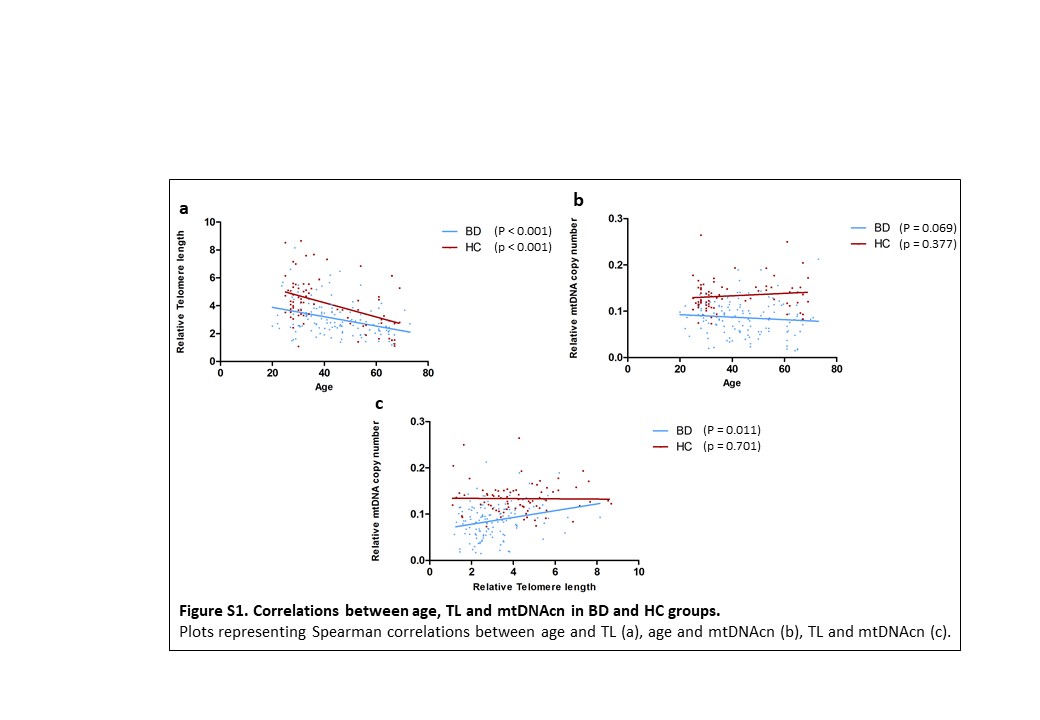

Supplement: Supplementary file 1 — Figure S1 [file 41398_2022_1891_MOESM1_ESM.jpg]
