# Supplementary material for: Telomere length and mitochondrial DNA copy number in bipolar disorder: identification of a subgroup of young individuals with accelerated cellular aging
Source: Transl Psychiatry. 2022 Apr 1;12:135. doi: 10.1038/s41398-022-01891-4 (PMC8975957; doi:10.1038/s41398-022-01891-4)
Supplement: Supplementary file 2 — Table S1 [file 41398_2022_1891_MOESM2_ESM.docx]

**Table S1. Factors associated with TL and mtDNAcn**

| **Predictor** | **TL** | |  | **mtDNAcn** | |
| --- | --- | --- | --- | --- | --- |
|  | **t** | **p** |  | **t** | **p** |
| Intercept | 7.997 | <0.001 |  | 6.231 | <0.001 |
| Age | -5.629 | <0.001 |  | -0.381 | 0.703 |
| Sex | 1.478 | 0.141 |  | 1.629 | 0.105 |
| BMI | -0.771 | 0.442 |  | 0.096 | 0.924 |
| Tobacco | 3.338 | 0.001 |  | 0.049 | 0.961 |
| MADRS | 1.323 | 0.187 |  | 0.808 | 0.420 |
| YMRS | -0.154 | 0.878 |  | 1.011 | 0.313 |
| BD (versus HC) | -5.448 | <0.001 |  | -8.390 | <0.001 |

Linear regression with either TL or mtDNAcn as dependent variables and clinical status (BD versus HC), age, sex, BMI, tobacco, MADRS and YMRS scores as independent variables.

BD Bipolar Disorder ; HC Healthy Controls ; BMI Body Mass Index ; MADRS Montgomery Asberg Depression Rating Scale ; YMRS Young Mania Rating Scale ; TL Telomere Length ; mtDNAcn mitochondrial DNA copy number.
