# Supplementary material for: Telomere length and mitochondrial DNA copy number in bipolar disorder: identification of a subgroup of young individuals with accelerated cellular aging
Source: Transl Psychiatry. 2022 Apr 1;12:135. doi: 10.1038/s41398-022-01891-4 (PMC8975957; doi:10.1038/s41398-022-01891-4)
Supplement: Supplementary file 3 — Table S2 [file 41398_2022_1891_MOESM3_ESM.docx]

**Table S2. Factors associated with clinical status (BD versus HC)**

| **Predictor** | **Clinical status** | | | |  | **Clinical status** | | | |
| --- | --- | --- | --- | --- | --- | --- | --- | --- | --- |
|  | **Estimate^a^** | **SE** | **Odds ratio** | **p** |  | **Estimate^a^** | **SE** | **Odds ratio** | **p** |
| Intercept | -1.265 | 1.70 | 0.28 | 0.456 |  | 0.199 | 1.99 | 1.22 | 0.920 |
| Age | -0.004 | 0.02 | 0.99 | 0.806 |  | 0.019 | 0.02 | 1.02 | 0.227 |
| Sex | 0.262 | 0.44 | 1.30 | 0.549 |  | 0.205 | 0.48 | 1.23 | 0.668 |
| BMI | 0.105 | 0.06 | 1.11 | 0.065 |  | 0.105 | 0.06 | 1.11 | 0.099 |
| Tobacco | 1.388 | 0.46 | 4.01 | 0.002 |  | 0.827 | 0.49 | 2.29 | 0.089 |
| MADRS | 0.955 | 0.22 | 2.60 | <0.001 |  | 0.904 | 0.22 | 2.47 | <0.001 |
| YMRS | 1.147 | 0.55 | 3.15 | 0.036 |  | 0.873 | 0.50 | 2.39 | 0.080 |
| TL | -0.653 | 0.19 | 0.52 | <0.001 |  | ̶ | ̶ | ̶ | ̶ |
| mtDNAcn | ̶ | ̶ | ̶ | ̶ |  | -41.34 | 7.61 | <0.001 | <0.001 |

Clinical status (BD versus HC) was used as the dependent variable, either TL or mtDNAcn as independent variables with an adjustment for age, sex, BMI, tobacco,

MADRS and YMRS scores.

BD Bipolar Disorder ; HC Healthy Controls ; BMI Body Mass Index ; MADRS Montgomery Asberg Depression Rating Scale ; YMRS Young Mania Rating Scale ;

TL Telomere Length ; mtDNAcn mitochondrial DNA copy number, SE Standard Error.

^a^ HC group was used as reference for logistic regression.
